# Pre-hospital portable monitoring of cerebral regional oxygen saturation (rSO_2_) in seven patients with out-of-hospital cardiac arrest

**DOI:** 10.1186/s13104-016-2239-4

**Published:** 2016-08-31

**Authors:** Tomoya Hirose, Tadahiko Shiozaki, Junji Nomura, Yasuto Hamada, Keiichi Sato, Kazuya Katsura, Naoki Ehara, Akinori Wakai, Kentaro Shimizu, Mitsuo Ohnishi, Sumito Hayashida, Daikai Sadamitsu, Takeshi Shimazu

**Affiliations:** 1 Department of Traumatology and Acute Critical Medicine, Osaka University Graduate School of Medicine, 2-15 Yamadaoka, Suita, Osaka 565-0871 Japan; 2 Chuo Fire Station, Osaka Municipal Fire Department, 2-1-6 Uchihonmachi, Chuo-Ku Osaka-City, Osaka 540-0026 Japan; 3 Traumatology and Critical Care Medical Center, National Hospital Organization Osaka National Hospital, 2-1-14 Hoenzaka, Chuo-Ku Osaka-City, Osaka 540-0006 Japan; 4 Osaka Municipal Fire Department, 1-12-54 Kujo-Minami, Nishi-Ku, Osaka-City, Osaka 550-8566 Japan

**Keywords:** Regional oxygen saturation, Out-of-hospital cardiac arrest, Pre-hospital, Emergency life-saving technician, Portable rSO_2_ monitor

## Abstract

**Background:**

In recent years, measurement of cerebral regional oxygen saturation (rSO_2_) has attracted attention during resuscitation. However, serial changes of cerebral rSO_2_ in pre-hospital settings are unclear. The objective of this study was to clarify serial changes in cerebral rSO_2_ of patients with out-of-hospital cardiac arrest (OHCA) in the pre-hospital setting.

**Methods:**

We recently developed a portable rSO_2_ monitor that is small (170 × 100 × 50 mm in size and 600 g in weight) enough to carry in pre-hospital settings. The sensor is attached to the patient’s forehead by the ELT (Emergency Life-saving Technician), and it monitors rSO_2_ continuously.

**Results:**

From June 2013 through August 2014, serial changes in cerebral rSO_2_ in seven patients were evaluated. According to the results of the serial changes in rSO_2_, four patterns of rSO_2_ change were found, as follows. Type 1: High rSO_2_ (around about 60 %) type (n = 1). Initial electrocardiogram was ventricular fibrillation and ROSC (return of spontaneous circulation) could be diagnosed in pre-hospital setting. Her outcome at discharge was Good Recovery (GR). Type 2: Low rSO_2_ (around about 45–50 %) type (n = 3). They did not get ROSC even once. Type 3: Gradually decreasing rSO_2_ type (n = 2): ROSC could be diagnosed in hospital, but not in pre-hospital setting. Their outcomes at discharge were not GR. Type 4: other type (n = 1). In this patient with ROSC when ELT started cerebral rSO_2_ measurement, cerebral rSO_2_ was 67.3 % at measurement start, it dropped gradually to 54.5 %, and then rose to 74.3 %. The cerebral oxygenation was impaired due to possible cardiac arrest again, and after that, ROSC led to the recovery of cerebral blood flow.

**Conclusion:**

We could measure serial changes in cerebral rSO_2_ in seven patients with OHCA in the pre-hospital setting. Our data suggest that pre-hospital monitoring of cerebral rSO_2_ might lead to a new resuscitation strategy.

**Electronic supplementary material:**

The online version of this article (doi:10.1186/s13104-016-2239-4) contains supplementary material, which is available to authorized users.

## Background

In recent years, measurement of cerebral regional oxygen saturation (rSO_2_) by near-infrared spectroscopy (NIRS) has attracted attention in many fields [1–4]. We thought that it might be useful for the development of a new resuscitation strategy. Some research groups have reported that cerebral rSO_2_ on hospital arrival can predict neurological outcome in patients with out-of-hospital cardiac arrest (OHCA) [5, 6], but we thought this might not be correct because the values of rSO_2_ always change depending on the patient’s situation at the time cerebral rSO_2_ is measured [7]. Some research groups have reported that serial changes of cerebral rSO_2_ in hospital cardiac arrest patients may reflect high-quality cardiopulmonary resuscitation (CPR) [8, 9]. We hypothesised that rather than measuring one rSO_2_ value at one time point, measurement of serial changes in the values of rSO_2_ would be important. To prove this hypothesis, pre-hospital measurement of rSO_2_ in patients was needed. We recently developed a portable rSO_2_ monitor that is small enough to carry in pre-hospital settings. In this study, we tried to detect the serial changes of rSO_2_ measured by the Emergency Life-saving Technician (ELT) in earlier phase after out-of-hospital cardiac arrest. The objective of this study was to clarify serial changes in cerebral rSO_2_ of patients with out-of-hospital cardiac arrest in the pre-hospital setting.

## Methods

### Study design and data collection

**Fig. 1:**
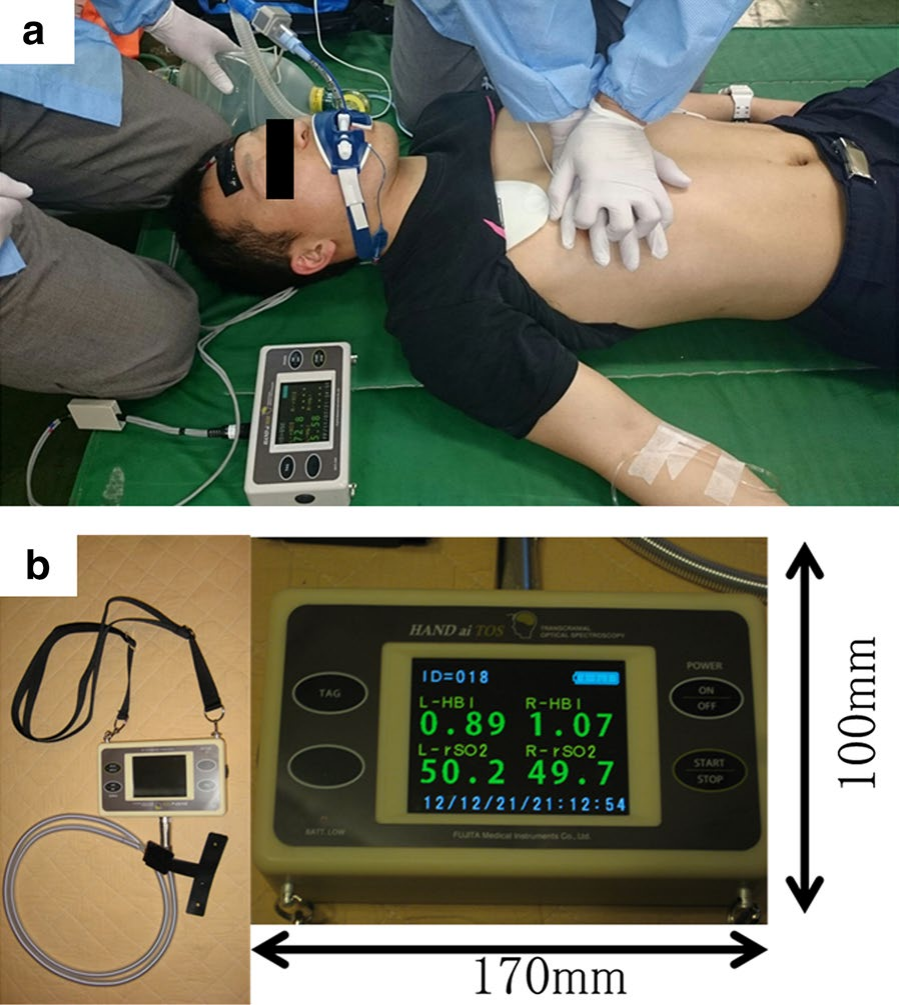
Photograph showing rSO_2_ measurement in a mock patient by ELTs and the portable near-infrared spectroscopy unit (portable rSO_2_ monitor). **a** The detector consists of two sensors that monitor the bilateral frontal lobes. The ELT can carry the portable rSO_2_ monitor (HAND ai TOS^®^; TOSTEC CO., Tokyo, Japan) and perform cardiopulmonary resuscitation without difficulty in the pre-hospital setting. **b** The portable rSO_2_ monitor is 170 × 100 × 50 mm in size and 600 g in weight. It can be carried easily by hanging it around the neck, and it can be used even in severe pre-hospital environments such as heavy rain and strong sunlight. *rSO*_*2*_ regional saturation of oxygen; *ELT* emergency life-saving technician

The subjects were all cardiopulmonary arrest (CPA) patients who were transferred to the National Hospital Organization Osaka National Hospital (Osaka, Japan) by ELTs based at Chuo Fire Station. The ELTs performed CPR according to recommendations of the Japan Resuscitation Council Guidelines 2010, which are based on the guidelines of the American Heart Association and the International Liaison Committee on Resuscitation [10]. The rSO_2_ sensor is attached to the patient’s forehead by the ELT (Fig. 1a). Medical staff did not change patient treatment according to rSO_2_ data.

The pre-hospital portable monitoring of cerebral rSO_2_ in CPA patients with OHCA was approved by the Ethics Committee of Osaka University Graduate School of Medicine (No. 12446), and the institutional review board waived the need for informed consent because the subjects were all in CPA.

### Portable NIRS rSO_2_ monitoring system

We developed a portable rSO_2_ monitor (HAND ai TOS^®^; TOSTEC CO., Tokyo, Japan) (Fig. 1b). The HAND ai TOS was not approved by Medicines and Healthcare Products Regulatory Agency (MHRA) and Food and Drug Administration (FDA). The HAND ai TOS system measures oxygen saturation based on the Beer-Lambert law by using three different wavelengths of near-infrared LED light, which have specific absorbance to oxyhaemoglobin and deoxyhaemoglobin. The lights pass through the skin to a depth of approximately 3 cm, and the reflected lights are sensed by a photodiode. The reflected lights represent the haemoglobin information mainly in the cerebral cortex. The system can measure rSO_2_ data every second without the necessity of arterial pulsation, so it is possible to carry out continuous monitoring in CPA patients. Two rSO_2_ values, left side and right side, are acquired continuously, and then the average of the two values is calculated. The normal range of cerebral rSO_2_ was previously determined from 15 healthy adult volunteers to be 71.2 ± 3.9 % (on room air) (n = 15, 10 men and 5 women, 43.2 ± 8.9 years) [7].

### Statistical analysis

All data are represented as mean ± standard deviation (SD). All statistical analyses were performed with JMP Pro 10 for Windows (SAS Institute Inc., Cary, NC, USA).

## Results

### Patient characteristics

From June 2013 through August 2014, serial changes in cerebral rSO_2_ in seven patients were evaluated. Characteristics, outcome, and rSO_2_ data of the OHCA patients are shown in Table 1. According to the results of the serial changes in rSO_2_, four patterns of rSO_2_ change were found, as follows.

**Table 1:** Characteristics, outcome, and rSO_2_ data of out-of-hospital cardiac arrest patients

No.	Age (years)	Witness	Bystander CPR	Initial ECG	ROSC	rSO_2_ at the start of measurement (%)	Outcome (GOS at discharge)
1	51	Yes	Yes	VF	Yes (pre-hospital)	60.1	GR
2	74	No	No	VF	Yes (after application of ECMO)	55.3	D
3	86	Yes	No	Unknown	Yes (before ELT contact)	66.1	VS
4	65	Yes	No	Asystole	Yes (in-hospital)	53.9	D
5	45	Yes	No	Asystole	No	45.3	D
6	27	No	Yes	Asystole	No	49.7	D
7	86	No	No	Asystole	No	52.9	D

*rSO*_*2*_ regional oxygen saturation, *CPR* cardiopulmonary resuscitation, *ECG* electrocardiogram, *ROSC* return of spontaneous circulation, *GOS* Glasgow outcome scale, *VF* ventricular fibrillation, *ECMO* extracorporeal membrane oxygenation, *ELT* emergency life-saving technician, *GR* good recovery, *VS* vegetative state, *D* death

### Type 1: high rSO_2_ type (Table 1; patient #1)

**Fig. 2:**
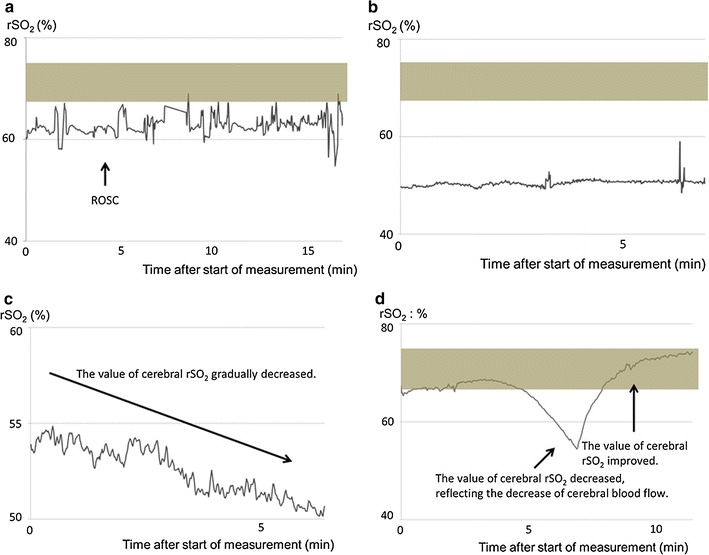
Serial changes in cerebral rSO_2_ (representative cases). **a** Type 1: High rSO_2_ type (around 60 %). One patient (51-year-old woman; patient #1) showed this type. Her initial electrocardiogram was ventricular fibrillation, and ROSC was diagnosed in the pre-hospital setting. Her outcome at discharge was good recovery. **b** Type 2: Low rSO_2_ type (around 45-50 %). Three patients (52.7 ± 30.2 years, 1 man and 2 women) showed this type. This* graph* shows the serial changes in cerebral rSO_2_ of patient #6. A similar pattern was observed in patients #5 and #7. None attained ROSC even once. **c** Type 3: gradually decreasing rSO_2_ type. Two patients (69.5 ± 6.4 years, 2 men) showed this type. Serial changes in cerebral rSO_2_ from patient #4 are shown. The rSO_2_ value gradually decreased. ROSC was diagnosed in-hospital 35 min after the start of measurement. His outcome was death. A similar pattern was also observed in patient #2. Both patients attained ROSC, which was diagnosed in hospital but not in the pre-hospital setting. Their outcomes at discharge were death. **d** Type 4: other type. One patient (86-year-old woman; patient #3) showed this type. In this patient with ROSC, when the ELT started cerebral rSO_2_ measurement, the cerebral rSO_2_ was 67.3 %. It dropped gradually to 54.5 % and then rose to 74.3 %. Cerebral oxygenation was impaired due to possible return of cardiac arrest, but after that, ROSC led to the recovery of cerebral blood flow. The *shaded area* represents the normal cerebral rSO_2_ range measured from healthy adults. *rSO*_*2*_ regional saturation of oxygen, *ROSC* return of spontaneous circulation, *ELT* emergency life-saving technician

Type 1 [high rSO_2_ type (n = 1)] is shown in Fig. 2a. The patient’s initial electrocardiogram (ECG) was ventricular fibrillation (VF). The rSO_2_ values remained at around 60 %. Return of spontaneous circulation (ROSC) was diagnosed 4 min after ELT contact in the pre-hospital setting. On hospital arrival, the patient’s consciousness was impaired (Glasgow Coma Scale [GCS], 1–2–5). Hypothermia therapy (34 °C) was performed for brain protection. Her outcome according to the Glasgow Outcome Scale (GOS) at discharge was good recovery. The etiology of CPA was considered cardiac arrhythmia.

### Type 2: low rSO_2_ type (Table 1; patients #5, 6, 7)

Type 2 (low rSO_2_ type [n = 3]) is shown in Fig. 2b (Table 1; patient #6). Her initial ECG was asystole. The rSO_2_ values remained at around 50 %. Her GOS outcome at discharge was death. A similar rSO_2_ pattern was observed in patients #5 and #7 (Table 1; Additional file 1: Figure S1B, C), whose ECGs also showed asystole. The mean value of cerebral rSO_2_ at the start of measurement was 49.3 ± 3.8 %. None of these patients attained ROSC even once.

### Type 3: gradually decreasing rSO_2_ type (Table 1; patients #2, 4)

Type 3 [gradually decreasing rSO_2_ type (n = 2)] is shown in Fig. 2c (Table 1; patient #4). This man’s initial ECG was asystole. His rSO_2_ values gradually decreased. ROSC was diagnosed in-hospital at 35 min after the start of measurement. His GOS outcome at discharge was death. A similar rSO_2_ pattern was observed in patient #2 (Table 1, Additional file 1: Figure S1A). ROSC was diagnosed in-hospital in patient #2 but not in the pre-hospital setting. The GOS outcome of patient #2 was also death.

### Type 4: other type (Table 1; #3)

Type 4 (other type [n = 1]) is shown in Fig. 2d. In this patient with ROSC when the ELTs started cerebral rSO_2_ measurement, cerebral rSO_2_ at measurement start was 67.3 % (almost normal range), it dropped gradually to 54.5 %, and it then rose to 74.3 %. Her cerebral oxygenation was impaired due to possible return of cardiac arrest, and after that, ROSC led to the recovery of cerebral blood flow. Her GOS outcome at discharge was vegetative state.

## Discussion

In this report, we showed the pre-hospital serial changes of cerebral rSO_2_ in patients with OHCA. The values of cerebral rSO_2_ in the patients with OHCA dramatically changed in the very early phase after cardiac arrest.

Our data showed several advantages of the pre-hospital measurement of cerebral rSO_2_. First, we might be able to predict the neurological outcome of patients with VF. The initial ECGs of patients #1 and #2 both showed VF, but the initial rSO_2_ value of patient #1 was higher than that of patient #2 (Table 1; Fig. 2a, c). In terms of neurological outcome, patient #1 experienced good recovery, whereas that of patient #2 was death (Table 1). Our report suggested that a patient who maintained a high cerebral rSO_2_ value might have a good neurological prognosis, and the type of serial change in the pre-hospital cerebral rSO_2_ data might lead to the prediction of neurological outcome in patients with VF.

Second, the pre-hospital cerebral rSO_2_ data might allow the estimation of the time after cardiac arrest in unwitnessed cases. Presence of a witness is one of the factors of good neurological prognosis [11, 12], but if witness information is absent, we might treat CPA patients as those for whom considerable time had passed after CPA. We could detect gradually decreasing cerebral rSO_2_ in two patients (Fig. 2c). We thought that the gradually decreasing rSO_2_ type probably indicated that little time had passed since CPA. To better understand the relation between time and cerebral rSO_2_, it will be necessary to accumulate data from more witnessed CPA patients.

Third, a portable rSO_2_ monitoring system would be very useful, similar to ECG monitoring, for ELTs in the pre-hospital setting. As shown in Fig. 2d, dynamic changes of cerebral rSO_2_ can be revealed. When the value of cerebral rSO_2_ decreased in Fig. 2d, the ECG showed QRS waves. We determined that pulseless electrical activity could be diagnosed at that time. The ELTs cannot always check the patient’s pulse in the pre-hospital setting, especially when they are transporting the patient on stairs or into the ambulance, because they must transfer the patient to hospital quickly. By using our portable rSO_2_ monitor, ELTs can always check cerebral blood flow without actually having to check the pulse.

Finally, the presence of a continuous low rSO_2_ type might predict poor neurological outcome and high mortality.

Our report has some limitations. First, rSO_2_ monitoring was not performed in a blind fashion. Therefore, rSO_2_ value may influence CPR procedures. Second, the validation of HAND ai TOS about low rSO_2_ value (<60 %) had been demonstrated in vitro but not in vivo. Third, sample size is small. To generalize this results, we need further study in a larger population.

## Conclusion

We could measure serial changes in cerebral rSO_2_ in seven patients with OHCA in the pre-hospital setting. Further evaluation of the validity of pre-hospital monitoring of cerebral rSO_2_ may lead to a new resuscitation strategy in the pre-hospital setting.

## Additional file

10.1186/s13104-016-2239-4 Serial changes in cerebral rSO_2_ (non-representative cases). Serial changes in cerebral rSO_2_ (non-representative cases). (A) 74-year-old man; patient #2. His initial electrocardiogram was ventricular fibrillation, and the values of cerebral rSO_2_ gradually decreased after start of measurement. ROSC was diagnosed in the hospital setting after application of ECMO. Her outcome at discharge was Dead. (B) 45-year-old woman; patient #5. This patient showed the low rSO_2_ type. None attained ROSC even once. (C) 86-year-old man; patient #7. This patient showed the low rSO_2_ type. None attained ROSC even once. rSO_2_, regional saturation of oxygen; ROSC, return of spontaneous circulation; ECMO: extracorporeal membrane oxygenation.

## Abbreviations

rSO_2_: regional oxygen saturation
ELT: emergency life-saving technician
ROSC: return of spontaneous circulation
OHCA: out-of-hospital cardiac arrest
NIRS: near-infrared spectroscopy
CPR: cardiopulmonary resuscitation
GOS: Glasgow outcome scale
GR: good recovery
VS: vegetative state
D: death
ECG: electrocardiogram
VF: ventricular fibrillation
GCS: Glasgow coma scale
ECMO: extracorporeal membrane oxygenation
PEA: pulseless electrical activity
