# Supplementary figures and images for: Pre-hospital portable monitoring of cerebral regional oxygen saturation (rSO2) in seven patients with out-of-hospital cardiac arrest
Source: BMC Res Notes. 2016 Aug 31;9(1):428. doi: 10.1186/s13104-016-2239-4 (PMC5007866; doi:10.1186/s13104-016-2239-4)

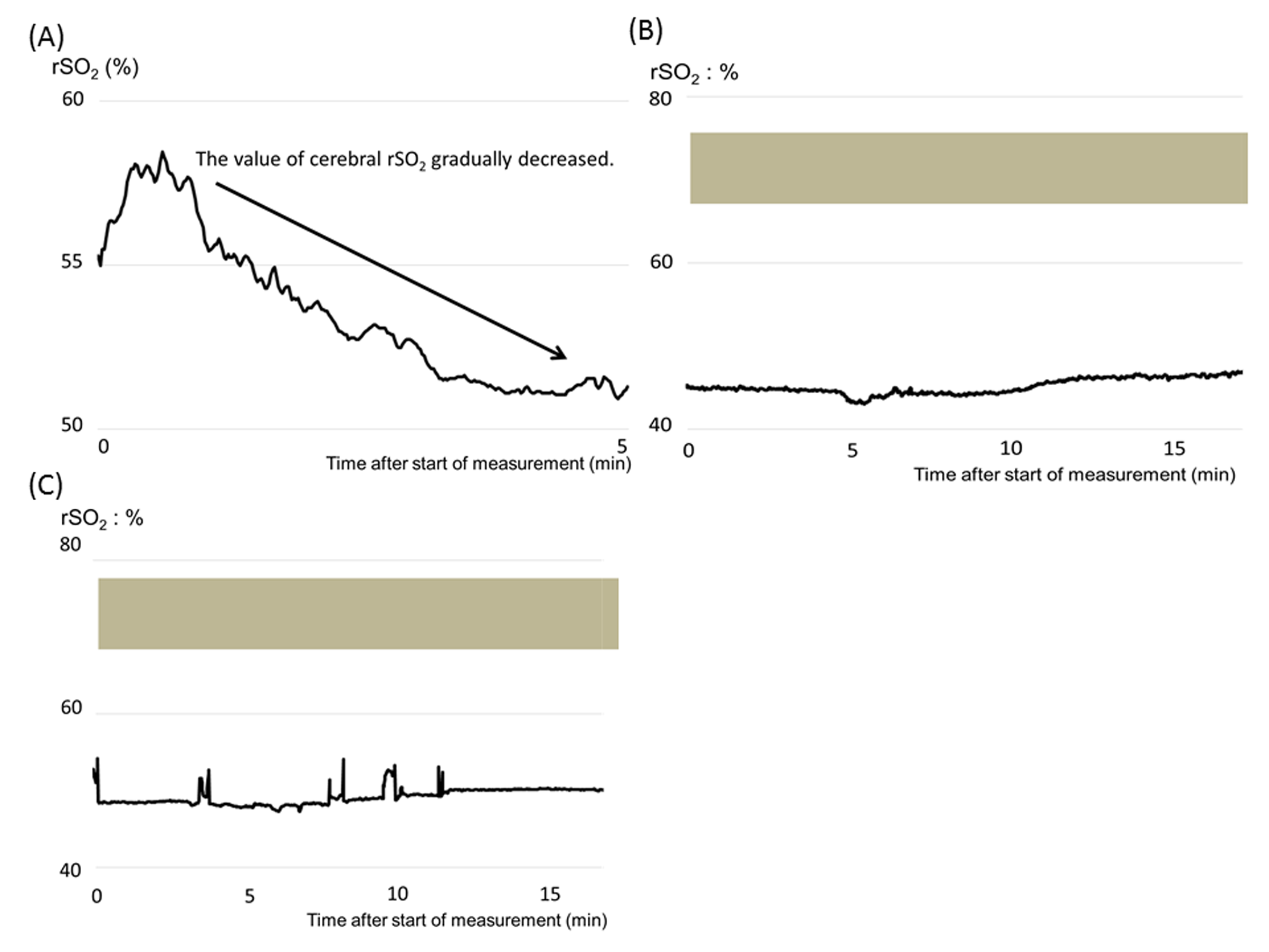

Supplement: Supplementary file 1 — 10.1186/s13104-016-2239-4 Serial changes in cerebral rSO2 (non-representative cases). Serial changes in cerebral rSO2 (non-representative cases). (A) 74-year-old man; patient #2. His initial electrocardiogram was ventricular fibrillation, and the values of cerebral rSO2 gradually decreased after start of measurement. ROSC was diagnosed in the hospital setting after application of ECMO. Her outcome at discharge was Dead. (B) 45-year-old woman; patient #5. This patient showed the low rSO2 type. None attained ROSC even once. (C) 86-year-old man; patient #7. This patient showed the low rSO2 type. None attained ROSC even once. rSO2, regional saturation of oxygen; ROSC, return of spontaneous circulation; ECMO: extracorporeal membrane oxygenation. [file 13104_2016_2239_MOESM1_ESM.tif]
